# Effect of Selected Silyl Groups on the Anticancer Activity of 3,4-Dibromo-5-Hydroxy-Furan-2(5*H*)-One Derivatives

**DOI:** 10.3390/ph14111079

**Published:** 2021-10-25

**Authors:** Radoslaw Kitel, Anna Byczek-Wyrostek, Katarzyna Hopko, Anna Kasprzycka, Krzysztof Walczak

**Affiliations:** 1 Department of Organic Chemistry, Chemistry Faculty, Jagiellonian University, Gronostajowa Street 2, 30-387 Krakow, Poland; radoslaw.kitel@uj.edu.pl; 2 Department of Organic Chemistry, Bioorganic Chemistry and Biotechnology, Faculty of Chemistry, Silesian University of Technology, Krzywoustego Street 4, 44-100 Gliwice, Poland; anna.byczek-wyrostek@polsl.pl (A.B.-W.); katarzyna.hopko@polsl.pl (K.H.); anna.kasprzycka@polsl.pl (A.K.); 3 Centre of Biotechnology, Silesian University of Technology, Krzywoustego Street 8, 44-100 Gliwice, Poland

**Keywords:** antiproliferative activity, apoptosis, furan-2(5*H*)-one, bioisosteres, silyl ethers

## Abstract

The pharmacological effects of carbon to silicon bioisosteric replacements have been widely explored in drug design and medicinal chemistry. Here, we present a systematic investigation of the impact of different silyl groups on the anticancer activity of mucobromic acid (MBA) bearing furan-2(*5H*)-one core. We describe a comprehensive characterization of obtained compounds with respect to their anticancer potency and selectivity towards cancer cells. All four novel compounds exert stronger antiproliferative activity than MBA. Moreover, **3b** induce apoptosis in colon cancer cell lines. A detailed investigation of the mechanism of action revealed that **3b** activity stems from the down-regulation of survivin and the activation of caspase-3. Furthermore, compound **3b** attenuates the clonogenic potential of HCT-116 cells. Interestingly, we also found that depending on the type of the silyl group, compound selectivity towards cancer cells could be precisely controlled. Collectively, we demonstrated the utility of silyl groups for adjusting both the potency and selectivity of silicon-containing compounds. These data reveal a link between the types of silyl group and compound potency, which could have bearings for the design of novel silicon-based anticancer drugs.

## 1. Introduction

With over 19 million new cases and 9.9 million deaths in 2020, cancer remains a worldwide cause of premature mortality [1]. Despite the continuous progress in the development of novel anticancer drugs, current treatments, including targeted therapies, have their limitations and they still cause severe side effects [2]. To overcome this, cancer immunotherapies have recently emerged to provide improved clinical outcomes. Disappointingly, not all cancers respond to such treatment and further evaluation of predictive markers and combination therapies is required before cancer immunotherapy becomes a gold standard in clinical oncology [3]. Therefore, there is still a great need for the discovery and development of new lead small-molecule compounds with increased activity and reduced toxicity to non-malignant cells. 

Furan-2(*5H*)-one scaffold and its variations are widely present in many bioactive natural products [4,5]. The modification of this structural motif led previously to multiple derivatives with a wide range of activity against cancers [6,7,8,9], bacterial infections [10,11], and fungus [12,13]. While the primary mechanism(s) of action and direct target(s) of furan-2(*5H*)-one remain unclear, some of its reported derivatives have been implicated in a range of functions including the inhibition of MDM2-p53 interaction [14], inhibition of COX-1 [15], as well as the inhibition of topoisomerase I [16]. In recent years, furan-2(*5H*)-one has emerged as a privileged scaffold in medicinal chemistry. The great interest in furan-2(*5H*)-one stems from two main reasons. First, this five-membered ring offers unique positioning of substituents thereby creating a scaffold that might be applied to design compounds to a wide range of molecular targets [17]. Second, the commercial availability of inexpensive reactive forms of furan-2(*5H*)-one, such as mucochloric (MCA) or mucobromic acids (MBA), provides medicinal chemists a rapid access for the generation of new molecules by tailoring the type of substituent and its position on the furan-2(*5H*)-one scaffold [18].

One of the main reasons for the failure of small molecules in in vivo and clinical studies is poor pharmacological and ADMET properties [19]. One potentially attractive strategy to improve the pharmacological properties of anticancer drugs is their modification with silicon-containing groups. Small molecules harboring hydroxyl groups offer great possibility to transform them into silyl derivatives, which may exhibit better pharmacological profiles and activity than the parent molecule. This is due to the unique properties of silicon, which have been reviewed previously in detail. Besides the bond length, the difference between carbon and silicon that is relevant in the drug design is the increased lipophilic character of silicon, which confers better penetration through cell membranes [20,21]. 

**Figure 1:**
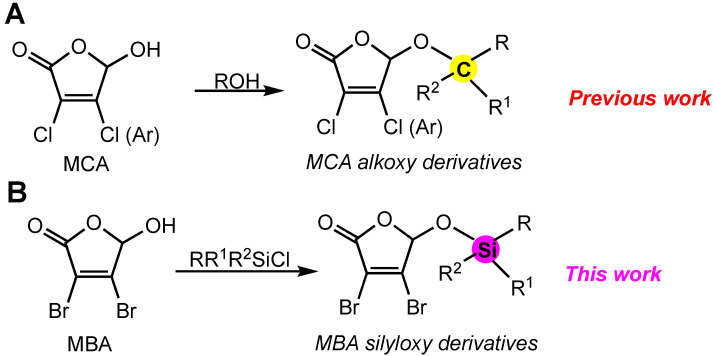
Strategy of compound design. (**A**) synthesis of alkoxy derivatives of MCA, (**B**) synthesis of silyloxy derivatives of MBA.

In our previous work, the design of mono- and disubstituted derivatives containing furan-2(5*H*)-one core was guided by the idea of incorporating aliphatic groups at C-5 and aromatic moieties at 4-C (Figure 1A). Primary SAR for 5-alkoxy derivatives of MCA suggests that the introduction of bulky hydrophobic groups at the C-5 position increases antiproliferative potency and provides a good level of selectivity towards non-small lung cancer cell line A549 [22]. In the current study, we hypothesized that silyl ethers of MBA may combine this effect with unique properties of silicon in terms of pharmacological properties. The concept is depicted in Figure 1B. The MBA molecule containing a reactive hydroxyl group is converted to its silyl ether derivative. This approach proceeds by reaction of the MBA with the silyl derivatizing agent. The nature of three auxiliary R and R1 groups would provide flexibility in modulation of both the hydrophobicity and hydrolytic stability of derivatives. 

Herein, we report the synthesis and the evaluation of the anticancer activity of novel set of MBA and its silyl ethers in several cancer cell lines. Interestingly, new derivatives were particularly active in colorectal cancer cell lines. Furthermore, the detailed investigation of the potential mechanism of action revealed that some of new silyl ethers of MBA have a complex mechanism of action which involves the down-regulation of survivin and caspase-dependent apoptosis.

## 2. Results

### 2.1. Chemistry

**Scheme 1:**
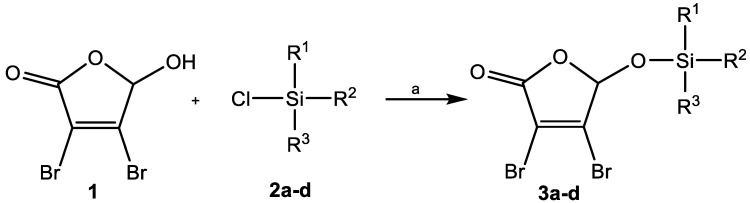
Synthesis of 5-substituted MBA derivatives. (a) DIPEA, DMF, 0 °C.

To access various 5-*O*-silylated MBA derivatives, commercially available 3,4-dibromo-5-hydroxyfuran-2(5*H*)-one (MBA) **1** was treated with different silyl chlorides **2a**–**d** (Scheme 1, Table 1). Silylation reactions were carried out in anhydrous DMF in the presence of DIPEA at 0 °C. This approach furnished compounds **3a**–**d** with yields ranging from 32% to 76%. The structure of the obtained MBA derivatives was confirmed using ^1^H NMR, ^13^C NMR and high-resolution electrospray ionization mass spectroscopy (ESI-MS).

### 2.2. Antiproliferative Activity

The in vitro antitumor activities of the MBA and its derivatives (Table 2) were assayed against a panel of human cancer cell lines (HCT116, HCT-116 ^−/−^p53, HT-29, MCF-7, SJSA-1, U2OS, HepG2 and Hep3B) using the MTT assay. As a reference drug we used 5-fluorouracil (5-FU). As illustrated in Table 2, three of four novel silyl derivatives of MBA displayed better antiproliferative activity than the parent compound in all tested cell lines. Interestingly, colon cancer cells were much more sensitive to MBA and its silyl derivatives than the cells of other types. In particular, compound **3a** and **3d** exhibited an excellent antiproliferative activity only in the HCT-116 cell line IC_50_ = 1.3 μM and 1.6 μM, respectively, while their efficacy in other tested cell lines was comparable to that of the MBA. In contrast, compounds **3b** and **3c** have been shown to be effective across all tested cancer cells with IC_50_ ranges 7.3 to 21.3 μM and 3.9 to 65.6 μM, respectively. Compound **3d** had superior anticancer activity in the HCT-116 and HCT-116 ^−/−^p35 cell lines, but was significantly less active in MCF-7 cells (IC_50_ = 89 μM) and therefore its evaluation in other cell lines has been halted. On the basis of in vitro antiproliferative activities, compounds **3b** and **3c** were selected for further mechanistic studies in the HCT-116 cell line. 

### 2.3. Cell Cycle Analysis and Apoptosis Induction

**Figure 2:**
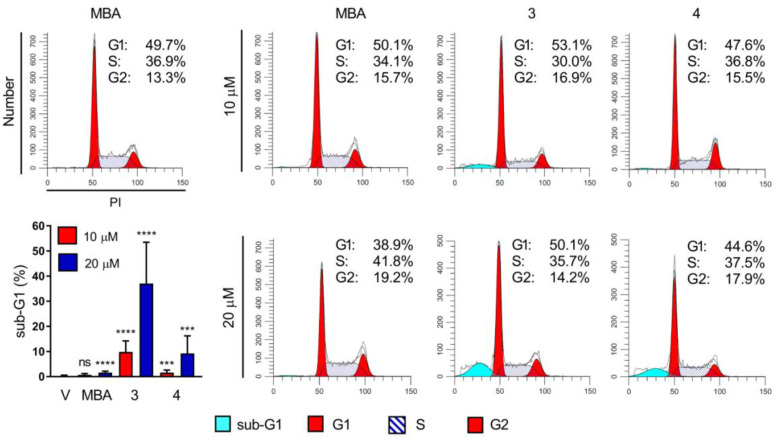
Effect of MBA and its silyl derivatives **3b** and **3c** on cell cycle in HCT 116 cells after 24 h exposure; Representative cytograms (with percentage of cells in cell cycle phases) from one of at least two independent experiments are shown; quantification of sub-G1 cell percentage is shown (left bottom column plot); ns—not significant, *** *p* < 0.005, **** *p* < 0.0005.

To better understand the origin of the higher susceptibility of colon cancer cells to MBA and its novel derivatives, compounds **3b** and **3c** were selected to test their ability to perturb the cell cycle in the HCT-116 cell line. Interestingly, neither the MBA nor its silyl derivatives **3b** and **3c** tested at 10 μM, influenced the progression of the cell cycle in HCT-116 after 24 h of exposure. When tested at 20 μM, the MBA decreased the number of cells in the G1 phase (from 49.7% to 38.9%) with a concomitant slight increase in the percentage of cells in both S and G2 phases. Compounds **3b** and **3c** did not alter the progression of the cell cycle at 20 μM as well. However, a significant increase in the percentage of sub-G1 cells after treatment with compounds **3b** and **3c** was observed. Compound **3b** increased the sub-G1 population by 10% and 31%, at a concentration of 10 μM and 20 μM, respectively, while compound **3c** exerts such effect only by 10% at the highest tested concentration (Figure 2). Of note, treatment with compound **1** did not cause any significant increase of the sub-G1 cells (0.7% at 10 and 1.6% at 20 μM) when compared to the untreated cells (0.3%). This result highlights the importance of the type of silyl group on the mechanism of action of tested compounds on HCT-116 cells. 

**Figure 3:**
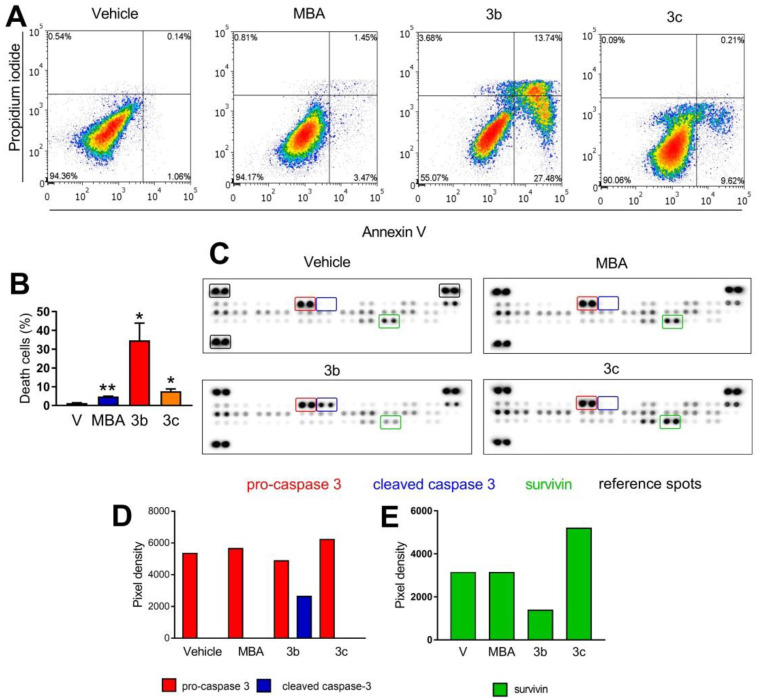
Effect of MBA and derivatives **3b** and **3c** on apoptisis in HCT-116 cell line after 24 h exposure at 20 μM. (**A**) representative cytograms (with percentage of cells in each quadrant) from one of at least two independent experiments are shown, (**B**) quantification of cell death as a sum of both annexin+/PI− and annexin+/PI+ cells, (**C**) changes in apoptosis-related proteins determined with using a Human Apoptosis Proteome Profiler kit. (**D**) quantification of changes of expression of pro-caspase-3 and cleaved caspase-3, (**E**) quantification of changes of expression of survivin; * *p* < 0.05, ** *p* < 0.005.

The concentration-dependent accumulation of cells in the sub-G1 peak after treatment with compounds **3b** and **3c** suggests that these compounds may exert their antiproliferative effects in HCT-116 through the induction of apoptosis. To verify this thesis, we utilized annexin-V/PI double staining of cells, which were subsequently subjected to flow cytometric analysis. The cells exposed to compound **3b** at 20 μM showed a significant increase in both early (annexin-V+/PI−) and late (annexin-V+/PI+) apoptotic populations, which accounts for roughly 30% of death cells in total (Figure 3A,B). The percentage of apoptotic cells after treatment with compound **3c** was lower and reached only 18.5%. In the same conditions, MBA tested at 20 μM had negligible proapoptotic activity. 

To explore in detail the molecular mechanism of apoptosis induction by compounds **3b** and **3c**, we employed Human Apoptosis Proteome Profiler arrays that allow quantification of apoptosis-related biomarkers (Figure 3C). We detected significant up-regulation of cleaved caspase-3, a well-known apoptosis marker, after short exposure (1 h) of HCT-116 to compound **3b** tested at 30.2 μM (2 × IC_50_). In such conditions, neither the MBA nor compound **3c** induced caspase-3 activation. Moreover, **3b** caused the down-regulation of survivin, a member of the inhibitors of apoptosis (IAP) family. Collectively, it can be concluded that silyl derivative **3b** promotes cell death in HCT 116 cells by the alteration of expression levels of survivin and activation of caspase-3.

### 2.4. Colony-Formation Assay

**Figure 4:**
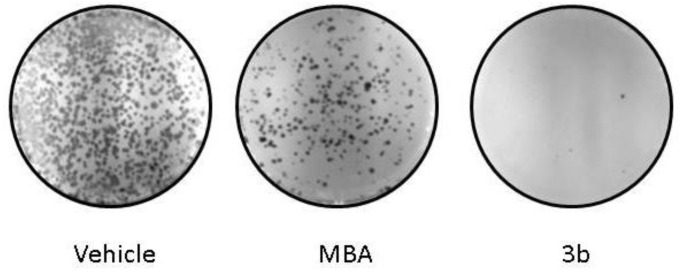
Silyl derivative **3b** abolishes the clonogenic potential of HCT 116 cells; Representative colony formation images of the HCT cells after 24 h treatment with MBA and **3b** are shown.

We next sought to investigate the effects of the MBA and compound **3b** clonogenic potential of the HCT-116 cell line. The cells were treated with compounds **1** and **3b** at 10 μM for 24 h. After harvesting, the cells were re-seeded and cultivated for another 10 days in a drug-free medium. Compound **3b** completely abolished the re-growth of HCT-116 colonies after 24 h of pretreatment. The MBA also attenuated the clonogenic potential of HCT-116 to a much less extent than the novel silyl derivative (Figure 4). 

### 2.5. Effect of Silyl Group on Compounds Selectivity

One of the major drawbacks of currently used anticancer drugs is their low selectivity, which often leads to undesired side effects that may be harmful to patients. Overcoming this challenging task requires special attention at the pre-clinical development level. To check whether silyl group incorporation may affect the selectivity of compounds towards cancer cells, MBA and all its new silyl derivatives were tested for their efficacy on noncancerous BEAS-2B and cancerous A549 cells line. The cytotoxicity of all silylated analogues in A549 cells was higher than that of the MBA (IC_50_ = 203 μM) exhibiting IC_50_ values ranging from 4.7 to 23.4 μM (Table 3). The MBA was also more cytotoxic to BEAS-2B cells with IC_50_ 39.7 μM and SI = 0.2, indicating that the parent compound affects cell viability in noncancerous cells more than in cancerous ones. Derivatives **3b** and **3c** were more potent than the MBA in the A549 cell line, but they also remarkably affect the viability of BEAS-2B cells (IC50 12.5 μM and 20.3 μM, respectively) having SI values 0.41 and 0.70, respectively. In contrast, compounds **3a** and **3d** were more cytotoxic to A549 cells with SI values 6.05 and 1.72, respectively. Strikingly, those two compounds have similar cLogP values 3.08 and 3.22, respectively. This suggests that incorporation of silyl groups into drugs may impact not only their efficacy but also could alter compound selectivity. 

## 3. Discussion

Targeting apoptosis is a promising strategy for cancer treatment. However, the number of FDA-approved drugs that exert proapoptotic activity is limited. Currently, multiple small-molecules triggering cancer cell death are under preclinical and clinical investigation. The majority of these agents act through the inhibition of anti-apoptotic members of the BCL-2 family (Bcl-2, Bcl-a, Mcl-2). Among them, only venetoclax gained FDA approval for the treatment of certain types of cancers. Therefore, there is an urgent need for the development of new therapeutic strategies that selectively target apoptosis in cancer. 

In a continuation of our efforts on the development of furan-2(5H)-one derivatives, we obtained here simple but potent compounds able to trigger colon cancer cell death. We found that their proapoptotic activity could be adjusted by the presence of particular silyl groups. This was especially highlighted by compounds **3a** and **3b**. The first one, bearing the TBDMS group was the most potent in suppressing cell proliferation (IC_50_ = 1.4 µM) but failed to induce apoptosis in the HCT-116 cell line. In contrast, compound **3b** having the TIPS group, rapidly triggered cell death in the HCT-116 cells. Why were there such dramatic differences in the proapoptotic activity of the silylated MBA derivatives? We speculate that silylation of the MBA may greatly improve penetration through cell membranes. The increase of proapoptotic activity through the incorporation of silyl groups (i.e., TBDMS) has been reported previously for several classes of compounds, including camptothecin and genistein glycoconjugates, and has been correlated with an increase of hydrophobicity [23,24]. In fact, the two most active compounds **3b** and **3c** have the highest cLogP values (4.06 and 4.68, respectively), a well-known indicator of cell permeability. This may suggest that among all tested compounds, **3b** and **3c** may be the most effective in the penetration of cell membranes. Another possible explanation of the superior proapoptotic activity of **3b** is that the bulky TIPS group enhances the interaction between the molecule and its intracellular target. Such potentiation by the introduction of silyl groups has been observed previously for compounds targeting HIV-1 reverse transcriptase (HIV-1 RT) [25,26].

We cannot also exclude that compounds developed by us may act as pro-drugs. It is well known, that depending on the steric hindrance on the silicon atom, the hydrolysis rates will vary significantly between derivatives. This would support our hypothesis that the TIPS group of **3b** enhances its interaction with molecular targets. However, further studies are required to identify direct molecular targets of tested compounds.

The mechanistic studies on the activity of compounds revealed that compound **3b** may act through targeting survivin, which ultimately leads to triggering apoptosis. 

## 4. Materials and Methods

### 4.1. Chemistry

All reagents were obtained from commercially available sources (Merck (Darmstadt, Germany), Sigma-Aldrich, and Avantor Performance Materials). NMR spectra were recorded on a Varian spectrometer 600 MHz and Agilent spectrometer 400 MHz in DMSO-d6 solution using tetramethylsilane (TMS) as an internal standard. Chemical shifts are reported as δ values (ppm). Melting point measurements were performed in an open capillary using Stuart^®^ SMP30 apparatus. High-resolution electrospray ionization mass spectroscopy (ESI-MS) experiments were performed using a Waters Xevo G2 QTOF instrument equipped with an injection system (cone voltage 50 V; source 120 °C).

#### Silyl Ethers of 3,4-Dibromo-5-hydroxy-furan-2(5*H*)-one **3a**–**d**

3,4-Dibromo-5-hydroxy-furan-2(5*H*)-one (1, 1 eq.) was dissolved in anhydrous DMF (10 mL DMF for 1 mmol of 1). The appropriate silyl chloride **2a**–**d** (1.1 eq.) and DIPEA (1.2 eq.) were added while stirring at 0 °C. The reaction mixture was stirred at 0 °C until TLC (MeOH:CHCl_3_, 5:95, *v*/*v*) indicated the total decay of the starting material (2 h). The reaction mixture was quenched with cold water (10 mL) and extracted with dichloromethane (2 × 10 mL). The organic layer was dried over anhydrous MgSO_4_ and the solvent was removed under reduced pressure. The residue was purified by column chromatography (n-hexane: ethyl acetate, 20:1 *v*/*v*) to give compounds **3a**–**d**.

##### 3,4-Dibromo-5-(*tert*-butyldimethylsilyloxy)-furan-2(5*H*)-one (**3a**)

Brown solid; yield: 76%; m.p. = 91–93 °C; ^1^H NMR (400 MHz, DMSO-d_6_) δ: 0.22 (s, 3H), 0.25 (s, 3H), 0.95 (s, 9H,), 5.99 (s, 1H, H-5); ^13^C NMR (100 MHz, DMSO-d_6_) δ: −5.25, −4.53, 18.00, 25.36, 98.77, 117.42, 146.18, 164.18; ESI-MS: *m*/*z* 392.9133 [M+Na^+^]^+^ (*m*/*z* calcd. 392.9133 [M+Na^+^]^+^).

##### 3,4-Dibromo-5-(triisopropylsilyloxy)-furan-2(5*H*)-one (**3b**)

Yellow oil; yield: 42%; ^1^H NMR (600 MHz, DMSO-d_6_) δ: 1.11 (s, 9H, 3×-CH_3_), 1.12 (s, 9H, 3×-CH_3_), 1.18–1.26 (m, 3H, 3×CH), 6.10 (s, 1H, H-5); ^13^C NMR (150 MHz, DMSO-d_6_) δ: 12.07, 17.65, 98.80, 117.61, 146.45, 164.33; ESI-MS: *m*/*z* 434.9602 [M+Na^+^]^+^ (*m*/*z* calcd. 434.9603 [M+Na^+^]^+^).

##### 3,4-Dibromo-5-(*tert*-butyldiphenylsilyloxy)-furan-2(5*H*)-one (**3c**)

Colorless oil; yield: 60%; ^1^H NMR (600 MHz, DMSO-d_6_) δ: 1.13 (s, 9H, C(CH_3_)_3_), 5.88 (s, 1H, H-5), 7.40–7.43 (m, 4H), 7.46–7.49 (m, 2H), 7.69–7.72 (m, 4H,); ^13^C NMR (150 MHz, DMSO-d_6_) δ: 19.41, 26.74, 96.55, 123.43, 149.95, 163.35; ESI-MS: *m*/*z* 516.9445 [M+Na^+^]^+^ (*m*/*z* calcd. 516.9446 [M+Na^+^]^+^).

##### 3,4-Dibromo-5-(trietylsilyloxy)-furan-2(5*H*)-one (**3d**)

Yellow oil; yield: 27%; ^1^H NMR (400 MHz, DMSO-d_6_) δ: 0.74 (q, 6H, J = 16.0 Hz, J^1^ = 8.0 Hz, 3 × Si-CH_2_-CH_3_), 1.02 (t, 9H, J = 16.0 Hz, J^1^ = 8.0 Hz, 3 × CH_3_), 6.00 (s, 1H, H-1); ^13^C NMR (100 MHz, DMSO-d_6_) δ: 4.72 (3 × CH_3_), 6.48 (3 × Si-CH_2_-CH_3_), 98.79, 117.58 (C3), 146.40 (C4), 164.35 (C2); ESI-MS: *m*/*z* 392.9134 [M+Na^+^]^+^ (*m*/*z* calcd. 392.9133 [M+Na^+^]^+^).

### 4.2. Cell Lines

Human cells lines: colon cancer (HCT-116 wt, HT-29), non-small lung carcinoma (A549), and bronchial epithelial cells (BEAS-2B) were obtained from American Type Culture Collection (ATCC, Manassas, VA, USA). Isogenic cell line HCT 116 p53^−/−^ (deleted both p53 alleles) were a gift from Dr. Bert Vogelstein. Other cell lines, namely MCF-7, SJSA-1, U2OS, HepG2, and Hep3B were received from the collection stored at Centre of Biotechnology of Silesian University of Technology. All cells were grown in DMEM with a high glucose medium (Sigma-Aldrich, Taufkirchen, Germany) supplemented with 10% (*v*/*v*) inactivated fetal bovine serum (FBS) (EURx, Gdańsk, Poland) and 1% antibiotics (10,000 μg/mL of streptomycin and 10,000 units/ml of penicillin) (Sigma-Aldrich, Taufkirchen, Germany) at 37 °C in humidified 5% CO_2_. The maximum concentration of DMSO in the culture media was 0.4%.

### 4.3. MTT Assay

5 × 10^3^ cells per well for the MCF-7 cell line and 2.5 × 10^3^ cells per well for the other cell lines were seeded in 96-well plates to adhere for 24 h. Afterward, the cells were treated with tested compounds. After 72 hours of incubation, 50 μL 0.5 mg/ml solution of MTT (3-(4,5-dimethylthiazol-2-yl)-2,5-diphenyl-2H-tetrazolium bromide) were added to each well and the plates were incubated for 3 h at 37 °C. Next, the MTT solution was removed from the plates and formed formazan crystals were dissolved in 2-propanol (Avantor Performance Materials, Gliwice, Poland) containing 0.04 M hydrochloric acid (Avantor Performance Materials, Gliwice, Poland). The absorbance was measured at 570 nm wavelength using a multiwall plate reader (BioTek, Winooski, VT, USA). The experiment was performed at a minimum of 3 independent replicates. Cell viability was set as a percentage versus vehicle control. IC_50_ was defined as a concentration of a drug that decreased cell viability by 50% relative to the untreated control.

### 4.4. Cell Cycle Analysis

The HCT 116 cells were seeded in 6-well plates (Nunc, ThermoFisher, Waltham, MA, USA) at a density of 5 × 10^4^ cells/well. After 24 h the medium was replaced with tested compounds. The cells were incubated for 24 h at 37 °C. Afterward, floating cells were collected and adherent cells were harvested by trypsinization and centrifuged at 600× *g*. The cells were washed with PBS (phosphate buffered saline) (Sigma-Aldrich, Taufkirchen, Germany) and then fixed in 70% ice-cold ethanol and stored at −20 °C overnight. The next day the cells were centrifuged at 600× *g*, washed with PBS, treated with 100 μg/mL RNase A solution (EURx, Gdańsk, Poland), 100 μg/ml solution of propidium iodide (PI) (Acros Organics, Geel, Belgium) and incubated for 30 min at room temperature in the dark. After staining, the cells were analyzed using a Becton Dickinson FACS Aria III sorter (BD Company, San Diego, CA, USA). Experiments were repeated at least twice. Data were analyzed using ModFit LTTM Software (Verity Software House, Topsham, ME, USA).

### 4.5. Annexin V-FITC Apoptosis Assay

The HCT 116 cells were seeded in 6-well plate (Nunc, ThermoFisher, Waltham, MA, USA) at a density of 5 × 10^4^ cells/well and allowed to attach. After 24 h the medium was replaced with tested compounds. The cells were then incubated for 24 h at 37 °C. Next, both floating and adherent cells were harvested and centrifuged at 600× *g*. The cells were washed with PBS (phosphate buffered saline) (Sigma-Aldrich, Taufkirchen, Germany) and we followed instructions from the manufacturer of the Annexin V-FITC Apoptosis Detection Kit (Biotool, Jupiter, FL, USA). Fluorescence was measured using a Becton Dickinson FACS Aria III sorter (BD Company, San Diego, CA, USA). Experiments were repeated at least twice. Data were analyzed using FCE Express 7 Software (DeNovo Software, Pasadena, CA, USA).

### 4.6. Analysis of the Expression of Apoptosis-Related Proteins

The Human Apoptosis Array Kit (R&D Systems, Abingdon, UK) was used to determine the levels of expression of apoptosis-related proteins. 2.5 × 10^5^ HCT 116 wt cells were seeded on 100 mm dishes (Nunc, ThermoFisher, Waltham, MA, USA) and allowed to attach for 24 h. Then the medium was replaced with tested compounds at a concentration corresponding 2 × IC_50_ value and the cells were incubated in 37 °C. After 60 min the cells were lysed and the lysates were clarified by centrifugation for 30 min at 14,000× *g*. The total protein concentration was measured using the Protein Quantification Kit-Rapid (Sigma-Aldrich, Taufkirchen, Germany) according to the manufacturer’s instruction. The next steps followed instructions from the manufacturer of the Human Apoptosis Array Kit. The membranes were then scanned in the G:Box trans illuminator (Syngene, Cambridge, UK) and the protein expression levels were quantified by densitometric analysis using ImageJ software. 

### 4.7. Clonogenic Assay

The HCT116 wt cells (5 × 10^4^ cells/well) were plated in 6-well plates (Nunc, ThermoFisher, Waltham, USA) and treated with the tested compounds for 24 and 72 h. Cells were harvested by trypsinization and then re-seeded on the new 6-well plate (Nunc, ThermoFisher, Waltham, MA, USA) with a density of 2 × 10^3^ cells/well. After 10 days the cells were washed with cold PBS (Sigma-Aldrich, Taufkirchen, Germany), fixed in ice-cold ethanol (−20 °C) for 3 minutes, air-dried, washed again with cold PBS and stained with 0.01% crystal violet (Sigma-Aldrich, Taufkirchen, Germany) in dH2O (deionized water) for 15 min. Next the plates were washed in dH_2_O and allowed to dry. Colonies were counted with a microscope (Zeiss, Oberkochen, Germany). Aggregates of 30 cells or more were considered as colonies. Photos were made using the G:Box transilluminator (Syngene, Cambridge, UK).

### 4.8. Statistical Analysis

The results were expressed as means ± S.D. and performed by unpaired *t*-test. All statistics calculations were carried out in GraphPad Prism software.

## 5. Conclusions

The strategy reported here provides the ability to control both hydrophobicity and the selectivity of novel derivatives of MBA. Silylation of 5-hydroxyl group in the MBA ring leads to set of novel compounds with increased cytotoxic potency against cancer cells in comparison to lead molecule **1**. Interestingly, compounds showed to be most active against colorectal cancer cell lines, which makes them potential candidates for the development of novel treatments for CRC. 

In conclusion, this study not only presents a set of potent and highly-cell-active compounds, but it also sheds light on the importance of silyl groups as crucial factors in addressing mechanisms of action and selectivity towards cancer cells. Taking into account the availability of various silylating agents, many further optimizations could be imagined in order to obtain compounds with an appropriate balance between potency and cancer cell selectivity. 

## Figures and Tables

**Table 1:** 5-*O*-Silyl ethers of MBA.

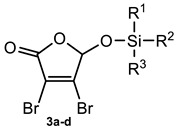			
Target Compound	R^1^	R^2^	R^3^
**3a**	Me	*tert*-Bu	Me
**3b**	*i*-Pr	*i*-Pr	i-Pr
**3c**	Ph	*tert*-Bu	Ph
**3d**	Et	Et	Et

**Table 2:** Cytotoxicity of MBA derivatives and 5FU against multiple cancer cell lines (nt—not tested).

	Compound					
Cell Line	MBA	3a	3b	3c	3d	5FU
HCT 116 wt	20.5 ± 5.2	1.3 ± 0.12	15.1 ± 1.2	6.3 ± 0.3	1.6 ± 0.4	6.3 ± 1.0
HCT 116 p53^−/−^	25.4 ± 6.0	21.4 ± 3.4	10.8 ± 1.0	4.0 ± 0.3	4.4 ± 0.4	13.4 ± 1.5
HT-29	97.1 ± 6.6	88.7 ± 8.2	7.3 ± 0.8	3.9 ± 0.2	nt	32.8 ± 4.7
MCF-7	141.8 ± 22.6	186.4 ± 14.2	7.7 ± 0.9	10.9 ± 0.7	89.2 ± 6.0	29.7 ± 1.4
SJSA-1	212.8 ± 8.8	196.3 ± 5.4	9.9 ± 1.1	14.9 ± 1.5	nt	nt
U2OS	97.6 ± 1.1	nt	9.3 ± 0.2	10.2 ± 0.8	nt	89.8 ± 2.1
HepG2	91.7 ± 2.2	135.4 ± 6.0	15.6 ± 1.2	nt	nt	nt
Hep3B	125.4 ± 10.5	135.3 ± 6.1	21.3 ± 3.5	65.6 ± 2.2	nt	nt

**Table 3:** Selectivity of MBA and compounds **3a**–**d** in NSCLC cell line A549 and normal bronchial epithelium cell line BEAS-2B.

Compound	Cell Line		SI	cLogP ^a^
	A549	BEAS-2B		
**MBA**	203 ± 0.3	39.7 ± 9.7	0.2	1.02
**3a**	23.4 ± 6.6	141.7 ± 23.1	6.05	3.08
**3b**	30.4 ± 4.2	12.5 ± 1.4	0.41	4.06
**3c**	29.1 ± 1.0	20.3 ± 0.6	0.70	4.68
**3d**	4.7 ± 0.1	8.1 ± 1.2	1.72	3.22

^a^—calculated with swissadme.ch.

## Data Availability

Data is contained within the article.
